# Quinpristin-Dalfopristin resistance among methicillin-resistant strains of staphylococci

**DOI:** 10.4103/0253-7613.62403

**Published:** 2010-02

**Authors:** S. Bhatawadekar, A. Chattopadhyay

**Affiliations:** Department of Microbiology, Bharati Vidyapeeth University Medical College, Pune, India E-mail: bsunita95@yahoo.com

I have read the case report “Emergence of Pristinamycin resistance in India” by Keshari *et al*[1] The authors have reported two cases of neonatal septicemia, caused by Methicillin-resistant *Staphylococcus aureus* (MRSA), showing primary *in vitro* pristinamycin resistance. We would like to share our observations with the readership of the IJP.

Methicillin resistant *Staphylococcus aureus* has become an important nosocomial pathogen. Glycopeptides have been the drugs of choice for treating MRSA infections. However, with the emergence of intermediate susceptibility to glycopeptides, other treatment alternatives, such as, quinpristin–dalfopristin and linezolid have become important. Quinpristin–dalfopristin is a combination of streptogramin B and A compounds with a synergistic activity against most gram-positive bacteria.[2] Quinpristin-dalfopristin is approved in the United States for treatment of infections caused by vancomycin-resistant strains of *E. faecium* and skin infections caused by MRSA and Streptococcus pyogenes. In Europe, it is also being used in the treatment of nosocomial pneumonia and other infections caused by MRSA.

We have isolated a total of 63 Staphylococci from blood cultures in the last six months. The blood cultures were processed by the automated and computerized blood culture system (BACTEC BD). The blood culture medium used was trypticase soya broth. Positive blood samples were subcultured and their growth was identified by standard biochemical reactions. Antibiotic sensitivity was conducted with the help of the API-BioMerieux machine. This instrument used a computer-assisted analysis of growth in plastic cards to calculate the minimum inhibition concentration (MIC). The ATB STAPH strip was designed following the National Committee for Clinical Laboratory Standards (NCCLS) / Clinical and Laboratory Standards Institute (CLSI) recommendations.[3–5]

Out of 38 *Staphylococcus aureus* isolated from the blood samples, 17 were MRSA. Three MRSA were found to be resistant to vancomycin (MIC > 16 mg/l), teicoplanin, and to quinpristin-dalfopristin (MIC > 2 mg/l). Out of the 17 MRSA, 14 showed sensitivity to vancomycin and quinpristin-dalfopristin. A total of 25 Coagulase negative staphylococci (CONS) were isolated from the blood samples. Out of nine methicillin-resistant CONS, four were detected to be vancomycin, teicoplanin, and quinpristin–dalfopristin resistant. All these seven vancomycin, quinpristin-dalfopristin-resistant staphylococci were isolated from the blood samples of neonates and pediatric patients. Out of these 63 isolates of staphylococci, 61 were found to be sensitive to minocycline. One interesting observation was that all the seven resistant isolates were sensitive to minocyclin [Table 1].

**Table 1:** Antibiotic resistance pattern of Staphylococci isolated from clinical samples

*Organisms*	*Number of Isolates*	*Methicillin resistant*	*Vancomycin / Teicoplanin*	*Quinpristin–Dalfopristin.*
*Staphylococcus aureus*	38	17	03	03
Coagulase negative	25	09	04	04
Staphylococci				

Although vancomycin and quinpristine–dalfopristin have been recommended as alternative treatments for MRSA, Staphylococci resistant to qinpristin–dalfopristin have been reported. Deep *et al*. studied the drug resistance of Staphylococci in north India.[6] They isolated 54% MRSA and 64% quinpristin–dalfopristin resistant *Staphylococcus aureus* from pus and urine samples. Four quinpristin–dalfopristin resistant CONS were also reported. Our findings are similar to your report. Quinpristin–dalfopristin is not used routinely in our hospital for treatment. Infections by gram-positive cocci are becoming more difficult to treat, because of the rapid emergence of antibiotic resistance. Many MRSA and MRCONS are found to be sensitive to quinpristin–dalfopristin. We also agree that more comprehensive region-wise laboratory work is needed, before advocating this drug for multidrug–resistant, gram-positive infections.

## References

[1] Keshari SS, Kapoor AK, Kastury N, Singh DK, Bhargava A (2009). Emergence of pristinamycin resistance in India. Indian J Pharmacol.

[2] Jones NJ, Ballow CH, Biedenbach DJ, Deinhart JA, Schentag JH (1998). Antimicrobial activity of quinupristin/dalfopristin (RP 59500, Synercid) tested against over 28,000 recent clinical isolates from 200 medical centers in the United States and Canada. Diagn Microb Infect Dis.

[3] National Committee for Clinical Laboratory Standards (2000). Performance standards for antimicrobial susceptibility testing. Twelfth Informational Supplement.

[4] Clinical and Laboratory Standards Institute (NCCLS) (2005). Performance standards for antimicrobial susceptibility testing. Fifteenth Informational Supplement.

[5] Clinical and Laboratory Standards Institute (NCCLS) (2008). Performance standards for antimicrobial susceptibility testing. Eighteenth Informational Supplement.

[6] Deep A, Goel N, Sikka R, Chaudhary U, Yadav S, Gupta A (2008). Quinpristin dalfopristin resistance in gram Positive bacteria: Experience from a tertiary care referral center in North India. J Infect Dis Antimicrob Agents.

